# Plant Vascular Tissues—Connecting Tissue Comes in All Shapes

**DOI:** 10.3390/plants7040109

**Published:** 2018-12-13

**Authors:** Eva Hellmann, Donghwi Ko, Raili Ruonala, Ykä Helariutta

**Affiliations:** 1 The Sainsbury Laboratory, University of Cambridge, Cambridge CB2 1LR, UK; eva.hellmann@slcu.cam.ac.uk (E.H.); donghwi.ko@slcu.cam.ac.uk (D.K.); raili.ruonala@slcu.cam.ac.uk (R.R.);; 2 Institute of Biotechnology, Department of Biological and Environmental Sciences, University of Helsinki, FI-00014 Helsinki, Finland

**Keywords:** Vasculature, Organogenesis, Development

## Abstract

For centuries, humans have grown and used structures based on vascular tissues in plants. One could imagine that life would have developed differently without wood as a resource for building material, paper, heating energy, or fuel and without edible tubers as a food source. In this review, we will summarise the status of research on *Arabidopsis thaliana* vascular development and subsequently focus on how this knowledge has been applied and expanded in research on the wood of trees and storage organs of crop plants. We will conclude with an outlook on interesting open questions and exciting new research opportunities in this growing and important field.

## 1. Vasculature and Its Arrangement

In the 19th century, the variety of vascular arrangements in form of different stele types attracted the interest of researchers. From their analyses, they could conclude that different forms of steles can specialize in supporting different functions and their different shapes are specific for plant groups, enabling them to draw phylogenetic connections between groups [1]. Even within one plant, various stele types occur. The different stele types vary not only with developmental stages, but also within different mature organs such as leaves, stem, hypocotyl, and roots. Although the structures in different species and organs are of diverse build, they share some of the underlying regulatory mechanisms and their main functions for the plant. Generally, they enable plants to transport water, nutrients, assimilates, as well as signalling molecules, and provide stability to the plant body. 

In this short review, we will focus on *Arabidopsis thaliana* as an example of an herbaceous species and as a commonly used model plant, in which many of the regulatory pathways for vascular development and arrangement have been elucidated. Furthermore, we will look at angiosperm trees, as they are a model for economically important wood production and tubers, which are essential agronomical food sources all over the world. As many processes underlying wood and tuber formation are shared, research on vascular development in *Arabidopsis* has and will inspire discoveries and development in economically and agronomically important vascular structures. Research on vascular development and expansion involving various species and growth forms is an excellent example of how basic research and applied research can work hand in hand to promote the growth of scientific knowledge and its application.

## 2. Vascular Development in *Arabidopsis thaliana*

Vascular development in *Arabidopsis thaliana* has been a topic of intensive research for decades. Basic principles of vascular development in roots, hypocotyl, leaves, and stems have been elucidated and gene regulatory networks have been inferred. In the following chapters, we will introduce the primary and secondary development of *Arabidopsis thaliana* root, hypocotyl, and stems, with its main regulators, and subsequently look at wood development and tuber formation.

### 2.1. Vascular Development in the Root

**Figure 1:**
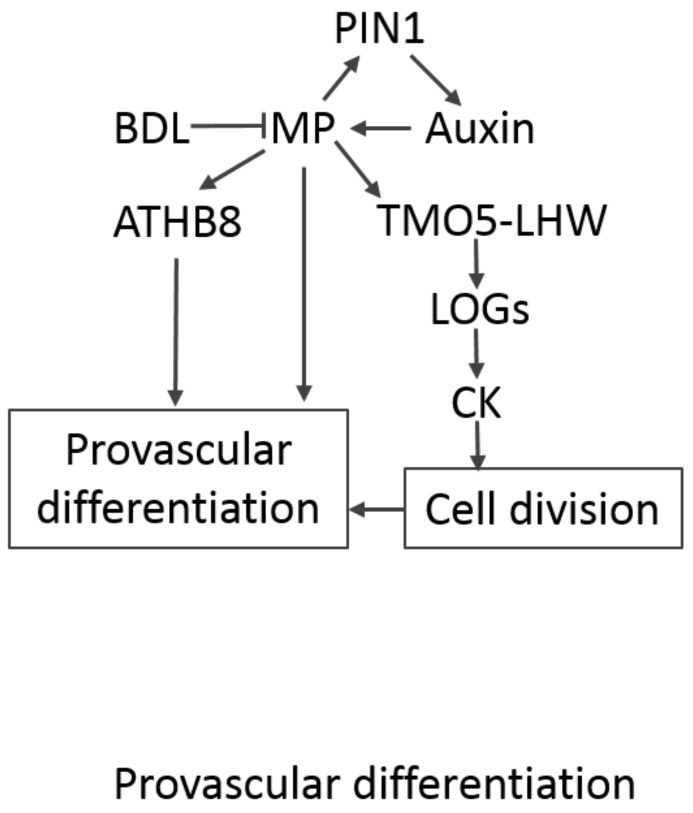
*Arabidopsis* provascular differentiation. MONOPTEROS (MP) is a central regulator in provascular development. It is induced by auxin and promotes auxin flow by induction of *PIN-FORMED (PINs)*. MP function is also modified by BODENLOS (BDL). MP enhances *ATHB8* expression, which contributes to provascular differentiation. It also regulates the TAGRET OF MONOPTEROS 5 (TMO5)–LONESOME HIGHWAY (LHW) dimer, which activates CK (cytokinin) biosynthesis and promotes cell division. LOG—LONELY GUY.

*Arabidopsis* root vascular development initiates during embryogenesis. Provascular tissue is specified by a spatially and temporally confined auxin maximum established by the PIN-FORMED (PIN) auxin transport function (Figure 1) [2,3]. *MONOPTEROS* (*MP*) expression, which marks future veins, is induced by auxin [4,5,6,7] and provides feedback on the auxin status by promoting *PIN1* expression [5,8,9,10]. Another component of auxin signalling, BODENLOS (BDL), was found to regulate *TAGRET OF MONOPTEROS* (*TMO*) *3*, *5*, *6*, and *7* upwards [10], which proved to be essential for proper MP function [11]. The MP–TMO5–LONESOME HIGHWAY (LHW) module, regulating cell division in the whole plant, was also found to play a role in the definition of the provasculature [8,11,12]. Among other factors, cytokinin is important for provascular development. The TMO5–LHW module induces cytokinin biosynthesis via activation of *LONELY GUY* (*LOG*) genes [13] and the cytokinin transporter PURINE PERMEASE 14 (PUP14) is required for early vascular development [14].

**Figure 2:**
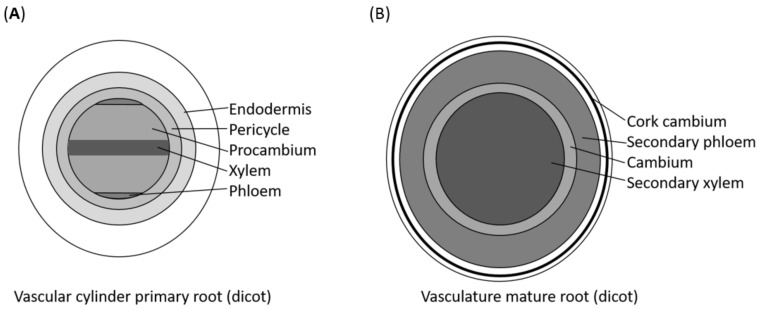
Schematic cross-sections of a primary root (**A**) and a mature root with secondary growth (**B**). In the primary root, two phloem poles are separated by procambium surrounding the central xylem axis. Around this structure, a ring of pericycle cells and endodermis cells can be found (**A**). In roots that have gone through secondary growth, there is a central secondary xylem cylinder surrounded by a continuous cambium and a ring of secondary phloem. Further out, a cork cambium can serve as a lateral meristem giving rise to cork and phelloderm (**B**).

**Figure 3:**
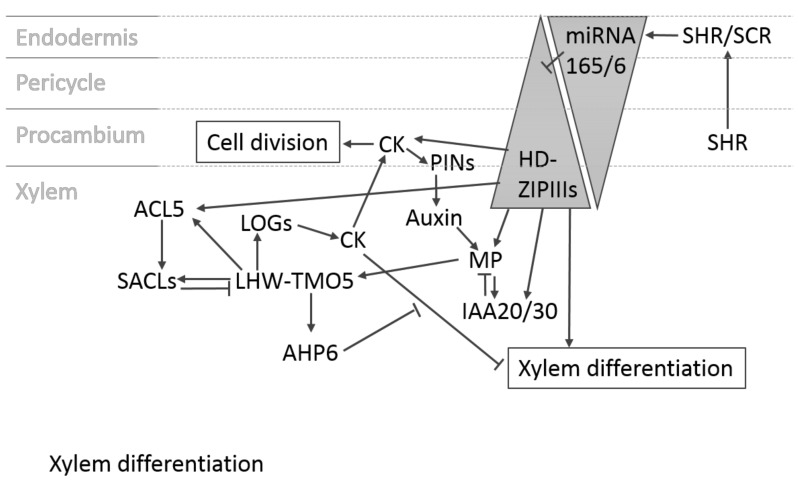
Xylem differentiation in the primary root. HD-ZIP IIIs are important promotors of xylem differentiation. Their level is regulated by a gradient of inhibitory miRNA165/6. miRNA165/6 levels are defined by SHORTROOT (SHR)/SCARECROW (SCR) dimer presence. The gradient is established by SHR diffusion from its production area in the procambium to the endodermis, where it forms the dimer with SCR that promotes miRNA165/6 expression. This results in a miRNA165/6 gradient with highest levels in the endodermis and an inverse gradient for HD-ZIP IIIs that promote xylem differentiation. The HD-ZIP IIIs induce *MP* and *IAA20/30*. They also enhance *ACL5* expression and CK (cytokinin) production. ACL5 induces translation of SUPRESSOR OF ACAULIS LIKE (SACL) genes that inhibit LHW–TMO5 dimerization and thus *LOG* expression, lowering the CK levels. The dimer also induces the CK signalling inhibitor *AHP6*, inhibiting the negative effect of CK on xylem differentiation. In the procambium, the CK inhibitory effects mediated by the HD-ZIP IIIs are not present, which leads to higher CK levels and signalling, resulting in cell division rather than xylem differentiation. CK induces PIN activity, pumping auxin out of the procambium. This causes an auxin maximum in the xylem axis, which subsequently induces *MP* expression.

The postembryonic root vasculature in *Arabidopsis* consists of a xylem strand that is surrounded by procambial cells and two opposing phloem poles. Layers of pericycle and endodermis enclose the vascular cylinder (Figure 2A). As is the case during embryogenesis, auxin and cytokinin play a major role in postembryonic development (Figure 3). Cytokinin reporters are expressed in the procambium, whereas auxin reporters mark the xylem cells [15,16]. The dominant negative cytokinin receptor mutant *wooden leg* (*wol*) shows a reduced number of vascular cell files and all inner cell types differentiate into protoxylem [17,18]. The lack of all three receptor kinases for cytokinin perception leads to a similar phenotype [19] as does the overexpression of a cytokinin degrading enzyme of the CYTOKININE OXIDASE (CKX) family [20,21]. The inhibitor of cytokinin signalling ARABIDOPSIS HISTDINE PHOSPHOTRANSFER PROTEIN 6 (AHP6) plays an important role in protoxylem differentiation [20]. It is upregulated by auxin and is a major component of the mutual inhibitory cytokinin–auxin feedback loop regulating procambium maintenance versus xylem differentiation [22]. Another interconnection between auxin and cytokinin regulation is the TMO5–LHW pathway. In postembryonic development, the TMO5–LHW dimer is, as in provascular development, induced by auxin via MP and activates cytokinin biosynthesis via upregulation of *LOG* genes [13]. Aside from auxin and cytokinin, phytohormone jasmonic acid has also been shown to regulate xylem development. An increase of jasmonic acid levels leads to extra xylem vessels, but this is abolished in jasmonic acid receptor mutants. Jasmonic acid function in vessel development is linked to cytokinin signalling via regulation of *AHP6* by the jasmonic acid regulated transcription factor MYC2 [23]. Further regulators of xylem differentiation include HD-ZIP IIIs that promote metaxylem development [24] and are regulated via the SHORTROOT (SHR)–SCARECROW (SCR) pathway [25,26] via the levels of the inhibitory miRNAs mi165/166 [24,27]. The metaxylem cell fate is also characterised by the expression of the thermospermine biosynthesis gene *ACAULIS 5* (*ACL5*) [28,29]. Thermospermine regulates the translation of the SUPRESSOR OF ACAULIS LIKE (SACL) protein family, which then affects the TMO5–LHW interaction that acts on xylem differentiation and cytokinin biosynthesis [12,13,30,31,32,33].

Protophloem differentiation in *Arabidopsis thaliana* is dependent on the OCTOPUS (OPS)–BRASSINOSTEROID INSENSITIVE 2 (BIN2)–BRASSINOSTEROID INSENSITIVE 1 (BIN1) cascade, on COTYLEDON VASCULAR PATTERN 2 (CVP2), and on the BREVIS RADIX (BRX)–BARELY ANY MERISTEM 3 (BAM3)–CLVATA3/ESR-related (CLE45) module. OPS represses *BIN2* [34,35]. BRX acts in a similar way and restricts *BAM3* expression confining CLE45 perception spatially [36,37]. Recently, other receptors for the CLE peptides have been identified that act independently of CORYNE (CRN)–CLAVATA 2 (CLV2). CLE-RESISTANT RECEPTOR KINASE (CLERK) and its homologues SENESCENCE-ASSOCIATED RECEPTOR-LIKE KINASE (SARK) and NSP-INTERACTING KINASE 1 (NIK1) represent a new module for CLE sensing in protophloem development [38,39]. The CLE45 signal was shown to be enhanced by MEMBRANE-ASSOCIATED KINASE REGULATOR 5 (MAKR5) action [40]. For sieve element differentiation, SUPPRESSOR OF MAX1-LIKE (SMXL) 3, 4, and 5 are required [41]. In contrast to the regulation of procambium proliferation and xylem differentiation, not cytokinin or auxin, but brassinosteroids are the most influential phytohormones for phloem differentiation [40,42,43]. Protophloem sieve element development is modulated by interaction with BRX and PROTEIN KINASE ASSOCIATED WITH BRX (PAX) with PIN1. Whereas BRX inhibits PIN1 mediated auxin efflux, PAX enhances it, leading to a balanced and ordered regulation of auxin distribution that is necessary for protophloem development [44]. 

ALTERED PHLOEM DEVELOPMENT (APL) regulates phloem differentiation [45,46]. NO APICAL MERISTEM, ATAF, CUP-SHAPED COTYLEDON (NACs), and NAC45/86-DEPENDENT EXONUCLEASE-DOMAIN PROTEINs (NENs) are involved in phloem maturation, which culminates in enucleation and the presence of fully developed sieve pores [47]. Furthermore, NAC20 was found to negatively regulate *APL* in phloem development [48].

Secondary growth in herbaceous dicotyledonous species such as *Arabidopsis* is characterised by the build-up of secondary cell walls in the xylem and lateral growth via a continuous cambium. These events are prominent in the *Arabidopsis* stem and hypocotyl, which are discussed next. 

### 2.2. Vascular Development in Shoot and Hypocotyl

The elongation of the *Arabidopsis* inflorescence stem (bolting) coincides with the transition from the vegetative to the reproductive stage. The primary shoot apical meristem is committed to producing flowers and the rib meristem is activated to push the newly forming flowers upwards from the vegetative rosette. The molecular mechanisms regulating the primary vascular patterning in the extending tip of the young stem are poorly understood [49]. The stem vasculature is organized in separate bundles that eventually become connected by a so-called interfascicular cambium [50]. In the basal part of the stem, in the vicinity of the rosette, the activity of the interfascicular cambium results in complete cylindrical rings of the vascular tissues: phloem, cambium, and xylem, one inside another. Like the primary vascular organization in the *Arabidopsis* root described in the previous section, the *Arabidopsis* hypocotyl (embryonic stem) develops a xylem axis in the centre of the stele and two phloem poles, which are intervened by procambial cells, during the primary growth [51,52]. Common molecular factors modulate the primary vascular development in the root and the hypocotyl. Mutants defected in the primary vascular patterning in the root also exhibit similar flaws in the hypocotyl vasculature. For instance, *MP* and *WOL* are expressed in the root and the hypocotyl vascular tissues during embryogenesis and post-embryonic development, and the mutants are impaired in the vascular patterning of both organs [6,18,53]. In contrast to the *Arabidopsis* root, which has been a representative system to study the primary growth, the *Arabidopsis* hypocotyl and inflorescence stem have been useful model systems to scrutinize the molecular processes underlying secondary growth [52,54,55]. Especially, the hypocotyl undergoes substantial secondary thickening by the activity of vascular cambium and cork cambium, similar to wood formation in trees. The hypocotyl does not grow longitudinally during secondary growth, which makes it easier to observe the progression of radial thickening in a time-dependent manner [52,56,57]. Indeed, multiple molecular components such as phytohormones, transcription factors, peptides, and receptors, orchestrating the secondary growth in the *Arabidopsis* hypocotyls and the inflorescence stem, have been characterised [52,54,55]. In this section, we will mainly introduce the signalling networks underlying the secondary development in the hypocotyl and the inflorescence stem.

The radial secondary growth of the hypocotyl starts after the cambium forms and can be divided into two distinct phases, characterised by the xylem expansion accompanied by a fibre differentiation [56,57,58]. In phase I, the early phase, xylem vessel elements emerge and the surrounding cells remain as xylem parenchyma cells [56]. Similarly, during the early phase in the phloem, sieve elements, companion cells and parenchyma cells differentiated, but not fibres [52]. The expansion rates of the two conducting tissues are comparable in the early stage; thereby leaving the proportions of xylem and phloem to the total transverse area of the hypocotyls roughly constant [57,58]. In contrast, in phase II, parenchyma cells in the xylem and the phloem differentiate into xylem or phloem fibres with thick secondary cell walls, providing mechanical strength to the plants. The xylem area expands faster than the phloem, which leads to a higher ratio of xylem to phloem, like wood [56,57,58] (Figure 2B). According to studies done by Ragni and co-workers [59], the transition from phase I to II in hypocotyls concurs with the development of the inflorescence stem (conversion from vegetative to reproductive growth) in various rosette plants including *Arabidopsis thaliana*, *Cardamine hirsute*, *Barberea verna*, and *Taraxacum officinalis* [57,59]. However, this seems to be characteristic to rosette plants as the non-rosette plants (*Arabis alpine*, *Aster alpinus*, *Nicotiana benthamiana*, and *Solanum lycopersicum*) examined undergo the xylem expansion during vegetative growth [59]. Ragni et al. also found that xylem expansion is not regulated by floral specification, bolting, or age of the plants, but by gibberellin (GA), a phytohormone that is produced in the shoot upon flowering induction [59]. The detailed molecular mechanism underlying the GA signalling-mediated fibre differentiation remains to be unveiled, but recently, it was reported that the GA increases the expression of *NAC SECONDARY WALL THICKENING PROMOTING FACTOR 1* (*NST1*) and *NST3*, the master transcription factors implicated in secondary cell wall thickening of xylem fibres [51,60]. They are homologous to the VND6 and VND7 factors, which are sufficient to guide secondary cell wall formation during xylem vessel formation [61]. In addition, it was shown that the leucine-rich receptor-like kinases (LRR-RLKs) ERECTA (ER) and its paralogue ER-LIKE1 (ERL1) prevent the premature GA-induced fibre differentiation in *Arabidopsis* hypocotyls upon the floral transition by suppressing the expression of *NST1* and *NST3* [51]. Not only GA-induced xylem fibre differentiation, but also the suppression of the two *NST*s by ER and ERL1 are largely dependent on the class I KNOTTED1-like homeobox (KNOX) transcription factor 1 (KNAT1)/BREVIPEDICELLUS (BP), which was previously shown to regulate xylem fibre differentiation in the inflorescence stem [51,62]. Furthermore, KNAT1/BP and another class I KNOX transcription factor, SHOOT MERISTEMLESS (STM), were shown to repress the transcription of *BLADE-ON-PETIOLE 1* (*BOP1*) and *BOP2*. Both encode BTB/POZ domain and ankyrin repeat-containing proteins, which negatively regulate xylem fibre differentiation in the hypocotyl [63]. Recently, Aurora kinases were identified as additional regulators of vascular development. They inhibit xylem and phloem formation via the transcriptional regulation of *ALTERED PHLOEM DEVELOPMENT* (*APL*), *VASCULAR-RELATED NAC-DOMAIN 6* (*VND6*), and *VND7* [64].

**Figure 4:**
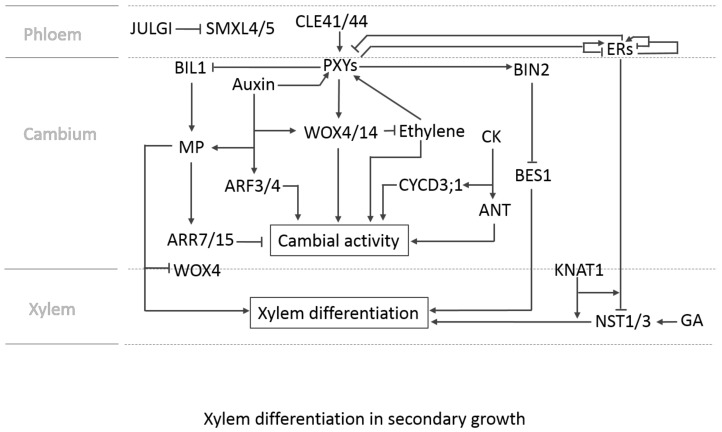
CLAVATA3/ESR-related (CLE)41/44/TRACHEARY ELEMENT DIFFERENTIATION INHIBITORY FACTOR (TDIF) generated in the phloem is perceived by the cambium-localised PHLOEM INTERCALATED WITH XYLEM (PXY)/TDIF RECEPTOR (TDR), which induces expression of *WUSCHEL-related HOMEOBOX (WOX)4*/*14* and activates BRASSINOSTEROID INSENSITIVE 2 (BIN2). WOX4/14 promotes cambial activity and BIN2 inhibits BRI1-EMS-SUPPRESSOR (BES1), which facilitates xylem differentiation. In addition, the CLE41/44/TDIF-PXY/TDR module enhances cambial activity by suppressing BIN2-LIKE 1 (BIL1)-mediated phosphorylation of MP that induces *ARR7*/*15* inhibition of cambial activity. The positive role of the auxin on cambium activity involves PXY and WOX4. In addition, auxin signalling upregulates the expression of *ARF3* and *ARF4* outside of the stem cell domain in the cambium, which facilitates the cambial proliferation. *MP* is induced by auxin and contributes to xylem differentiation via repressing *WOX4* but activating xylem-related genes in the cambium. Ethylene induces the expression of *PXY* and *ERF109*, *ERF018*, and *ERF1*, which enhances the cambial activity. WOX4 suppresses ethylene signalling. Cytokinin upregulates the expression of the D-type cyclin *CYCD3;1* and *AINTEGUMENTA* (*ANT)* to enhance cambial activity [86]. Gibberellin (GA) signalling facilitates xylem fibre differentiation by elevating the expression of *NST1* and *NST3* in a *KNAT1*/*BP*-dependent manner. In contrast, ERECTA (ER) and ER-LIKE1 (ERL1) inhibit the expression of *NST1* and *NST3* in a *KNAT1*/*BP*-dependent manner and suppress xylem differentiation. The two families of leucine-rich receptor-like kinases (LRR-RLKs), PXYs (PXY, PXL1, PXL2) and ERs (ER, ERL1, ERL2), mutually regulate their expressions. In the stem, ER suppresses the expression of *PXL1* and *PXL2*, whereas PXYs and ER upregulate the expression of *ERL1* and *ERL2.* However, in the hypocotyl, PXYs and ER repress the expression of *ERL1* and *ERL2.* Please note that we describe ER in the phloem section of the figure for simplicity, but it was shown that ER is expressed in the epidermis, phloem, and xylem of inflorescence stems [87], and ER and ERL1 are expressed in the stele of hypocotyls [51]. JULGI, which is expressed in the phloem and cambium, inhibits translation of SUPPRESSOR OF MAX1-LIKE (SMXL)4/5 by binding to the 5’ UTR of their mRNAs, and thereby suppresses phloem differentiation.

In addition to the genetic interactions implicated in fibre differentiation, a few other transcription factors involved in the cambial activity in hypocotyls and stems have been identified (Figure 4). For instance, *WUSHEL-related HOMEOBOX 4* (*WOX4*) and *WOX14* are upregulated by the CLE41/44/TRACHEARY ELEMENT DIFFERENTIATION INHIBITORY FACTOR (TDIF) (peptide ligands)-PHLOEM INTERCALATED WITH XYLEM (PXY)/TDIF RECEPTOR (TDR) (LRR-RLK) module in the cambium and play a part in cambial proliferation [65,66,67,68,69,70]. In parallel to the CLE41/44/TDIF-PXY/TDR module, the signalling by the phytohormone ethylene facilitates cambial cell division by inducing *ETHYLENE RESPONSE FACTOR*s (*ERF*s), such as *ERF109*, *ERF018*, and *ERF1* [71]. It was suggested that the two signal cascades interact with each other via ethylene, inducing the expression of *PXY/TDR* but WOX4 suppressing ethylene signalling [71]. Two more receptor-like kinases, REDUCED IN LATERAL GROWTH1 (RUL1) and MORE LATERAL GROWTH1 (MOL1), are also involved in regulation of cambial activity [72,73]. There seem to be complex interactions between hormonal pathways, the LRR-RLKs and the transcription factors to fine-tune vascular development. For example, *WOX4* is also shown to be upregulated by auxin and the induction is stabilized in a PXY/TDR-dependent manner [74]. Recently, it was reported that WOX14 is also involved in the xylem differentiation by inducing the expression of GA3-oxidase, which catalyses the production of bioactive GAs in the vascular bundle of the inflorescence stem [75]. Furthermore, in the stem, ER is shown to suppress the expression of *PXY-LIKE 1* (*PXL1*) and *PXL2*, while PXY, PXL1, PXL2, and ER upregulate the expression of *ERL1* and *ERL2* [76]. Interestingly, the interactions in the hypocotyl are distinct from those in the stem. In the hypocotyl, PXY, PXL1, PXL2, and ER repress the expression of *ERL1* and *ERL2* [76].

Furthermore, other phytohormones, such as auxin, cytokinin, strigolactone, and jasmonic acid, positively regulate cambial activity [77,78,79] and the interactions between key regulators during the secondary growth were recently analysed by network modelling [80]. Recently, the molecular interactions between auxin, cytokinin, and PXY signalling have been elucidated. Han and co-workers demonstrated that the CLE41/44/TDIF-PXY/TDR module regulates cambial proliferation by inhibiting BIN2-LIKE 1 (BIL1). BIL1 phosphorylates MP, which, upon phosphorylation, enhances the expression of *ARABIDOPSIS RESPONSE REGULATOR (ARR) 7* and *15*, resulting in suppression of cambial activity [81]. Moreover, it was reported that auxin signalling in the *Arabidopsis* inflorescence stem not only promotes cambial activity by inducing *AUXIN RESPONSE FACTOR (ARF) 3* and *4* expression outside of the stem cell domain in the cambium, but also facilitates xylem differentiation of cambial cells through MP suppression of *WOX4* activity and direct activation of xylem-related genes [82]. Interestingly, *WOX4* expression is not altered in the *bil1* mutant, suggesting that the suppression of *WOX4* by MP would be independent of the BIL1-mediated phosphorylation [81]. In addition to promoting the cambial proliferation, the CLE41/44/TDIF-PXY/TDR module also represses xylem differentiation of cambial cells by stimulating the activity of BIN2. BIN2 inhibits *BRI1-EMS-SUPPRESSOR* (*BES1*), a downstream transcription factor of brassinosteroid signalling [83]. Not much is known about upstream acting factors, but it was shown that *KANADI* genes, GARP family transcription factors, negatively regulate cambial activity by disrupting expression and polar localization of PIN1 [84]. More recently, a novel regulator involved in phloem differentiation has been characterised. The zinc-finger RNA-binding protein JULGI binds to the 5’ UTR of SMXL4/5 mRNA, inhibiting their translation and suppressing phloem development [85]. 

## 3. Agronomically Important Structures Derived from Plant Vasculatures

As summarised above, a substantial amount of knowledge has been gained by examining vascular development in *Arabidopsis*. In the next two sections, we will focus on how this knowledge can and has been applied to agronomically important plants, especially to wood producing trees, and to species that produce edible tubers as storage organs. On the other hand, research in these fields has provided new insight that is feeding back into research on *Arabidopsis.*

### 3.1. Wood Development—Secondary Growth of Trees

Spontaneously, one might not consider *Arabidopsis*, a small inconspicuous weed, to be beneficial for studies on secondary growth. However, at a miniature scale, many developmental events found in *Arabidopsis* can mimic the same principal features that are landmarks for trees, even down to a molecular level. One such event characteristic of trees is the extensive formation of woody tissues in the trunk. A multitude of factors, for example, cytokinin, auxin, gibberellin and ethylene, HD-ZIP IIIs, as well as the PXY-CLE41/44 signalling pathway and its target *WOX4*, have been shown to influence secondary growth in trees [88,89,90,91,92,93,94,95] in a manner similar to *Arabidopsis*. These aspects have been extensively reviewed (e.g., [96,97,98]; also, see above). In this section, we provide an overview of wood (secondary xylem) characteristics in angiosperm trees, and highlight some recent advances in this research field. 

**Figure 5:**
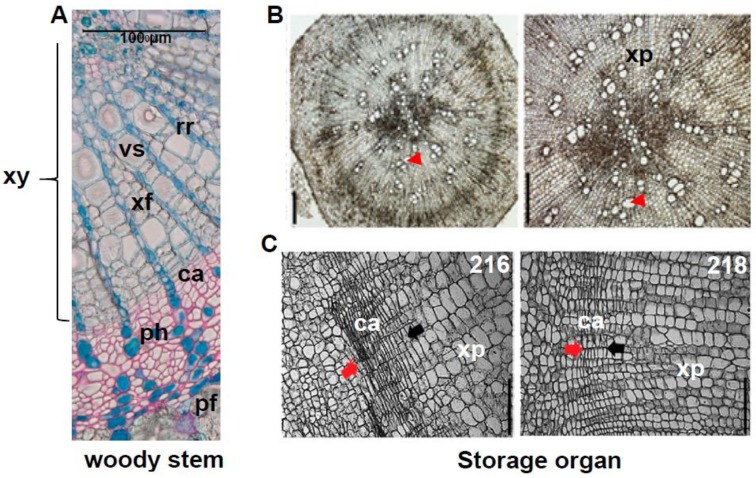
Cross-sections of birch (*Betula pendula*) stem (**A**) and storage organs of radish (*Raphanus sativus)* (**B**,**C**). Angiosperm wood is composed of xylem fibres and vessels to a large extent (**A**), while there is a substantial number of xylem parenchyma cells in the radish of three-week-old line 216 (**B**). The red arrow head indicates one of the xylem vessel cells. The size bar is 200 µm (**B**). The line 216 (**left**), which generates the larger storage organ, harbours a wider cambial zone when compared with the line 218 (**right**). Cambium zones are marked by red and black arrows with the red arrows on the side of the cortex region. The size bar is 100 µm (**C**). Abbreviations: xy—xylem; vs—vessel; xf—xylem fibre; rr—radial ray; ca—cambium; ph—phloem; pf—phloem fibre; xp—xylem parenchyma. (**A**) By courtesy of Chang Su, University of Helsinki; (**B**) and (**C**) adapted with permission from Jang et al. (2015) [106] and http://www.biologists.com/journal-of-experimental-biology/ doi:10.1093/jxb/erv220.

Secondary growth relies on closely coordinated cell division in the meristematic zone (the cambium); subsequent expansion; secondary cell wall development; and, in some cases, programmed cell death, all of which finally result in differentiated daughter cells serving their function. In a tree trunk during the active growth season, the cambial zone is composed of several layers of thin-walled cells that appear alike in histological cross-sections (Figure 5A). Recently, Bossinger et al. [99] performed an interesting somatic sector analysis in the *Populus* stem, suggesting the existence of a single cell layer of cambial initials, thought of as stem cells, that can divide in both anticlinal and periclinal orientations, and independently give rise to xylem or phloem. With their system, the authors succeeded in visualizing cell fate during wood development deep inside the trunk over the course of several months, providing insight into the cambial dynamics in a mature tree trunk. Another recently reported toolkit that may be expected to advance our understanding of wood development is the protein–protein and protein–DNA interactome, covering a set of genes expressed in the secondary tissues of *Populus* trunk [100,101]. On top of the high-resolution transcriptomics, hormonal profiling, and proteomics data accumulating from *Populus* ([102,103,104,105]), this adds to the growing body of resources available from this prominent tree model species.

Cambium produces secondary xylem, wood, towards the pith of the stem. Wood appears heterogeneous in a sense that it is composed of several cell types with a variable size and function, however, the majority of them are hollow and heavily lignified when mature ([107]). Besides lignin, cellulose and hemicellulose are major components of the secondary cell wall [108,109]. Such solid structures are necessary to support the weight of the plant tissues, including various substances within these tissues, as well as to provide protection against parasites and bacteria. The water-conducting cells are commonly known as tracheary elements (vessels and tracheids). Of these, vessels are the primary conduits for long-distance water transport in the angiosperm wood, while tracheids are predominant in gymnosperms. Typically, vessel elements are decorated by secondary cell wall thickenings and connected at their ends by perforated cell plates to allow a continuum throughout the plant. Vessels are outstanding by terms of a large diameter when compared with any other xylem cell type, which contributes to high efficiency in water transport. On the other hand, the width of the vessels increases the risk of embolism induced by freeze–thaw cycling at temperate regions or during drought (see [110]). Correlations between embolism resistance and lignin contents of wood have been indicated, suggesting that both the herbaceous, including *Arabidopsis*, and tree species with a high lignin content are more resistant to embolism [111,112]. Factors underlying the spatial patterning of vessels, or any other cell type, within the wood are poorly understood, however, a recent report suggests a role for basipetal auxin transport in *Populus* vessel distribution [113]. 

In addition to the vessels, wood contains two other cell types: fibres and parenchyma. The thick-walled fibres constitute the bulk, up to 80% of the angiosperm wood (commonly named hardwood) volume [110] and provide mechanical support to the plant. In *Arabidopsis*, xylem fibres are found in all organs undergoing secondary growth upon induction of flowering. The hormonal and molecular basis of vessel and fibre differentiation processes has been studied extensively in various plant systems such as *Arabidopsis* and *Populus*, and important discoveries regarding secondary cell wall formation and programmed cell death have also arisen from *Zinnia* and *Arabidopsis* suspension cell culture systems. In previous sections, we discussed some factors involved in fibre and vessel differentiation, however, further perspectives on this topic are provided in a number of recent reviews (e.g., [107,108]). While the vessels and fibres are programmed to die, the xylem parenchyma remain as living cells. In trees, parenchyma cells form rays that facilitate radial transport of water and solutes across the vascular tissues. Furthermore, rays function in carbohydrate storage and protection from embolism within the xylem [114]. As the secondary growth in *Arabidopsis* is limited, rays apparently do not develop spontaneously and seem to represent a rare aspect of wood development that, in addition to seasonality, requires a long-living woody species for functional studies. However, formation of ray-like cells has been reported in *Arabidopsis* stems, where secondary growth was induced under weight stress [115], further highlighting the amenable nature of this little weed for a wide array of manipulations. 

It is notable that wood is porous yet stiff, and typically requires drying as well as chemical processing prior to use as a construction material or pulp [116]. Wood processing methods are constantly optimized; for example, Song et al. [117] reported a compression method that, in combination with a carefully designed chemical treatment to partially remove lignin and hemicellulose, increased wood stiffness and strength by an impressive factor of 11. The authors were able to increase the strength of the cellulose component and, in fact, modify the wood structure and composition such that one might draw an imaginary analogue to tension wood (dried and flattened to an extreme). In nature, tension wood develops in the upper side of a tree branch or as a result of bending, to support the weight of the leaning structure. When compared with normal wood, tension wood appears more compact with smaller cell sizes. Furthermore, the cellulose content is higher and the mechanical strength is increased in this special type of angiosperm wood. Various phytohormones, including auxin, gibberellin and ethylene, have been implicated in the formation of tension wood-like features and transcriptomics analyses in *Populus* have shed light on the molecular control of this phenomenon (reviewed by Groover [118]). In the next sections, we move on to different types of special structures, edible storage organs, which, however, are also composed of vascular tissues. 

### 3.2. Tubers—Edible Storage Organs

Various plant species have evolved secondary growth mechanisms specialized to produce storage organs composed of many parenchyma cells that accumulate photosynthates in the form of sucrose or starch. For instance, *Raphanus sativus* (radish), *Brassica rapa* (turnip), *Daucus carota* (carrot), and *Beta vulgaris* (sugar beet) generate storage organs (tubers) from their taproots. *Manihot esculenta* (cassava) and *Ipomoea batatas* (sweet potato) produce them from their fibrous roots and *Solanum tuberosum* (potato) forms tubers from stolons, underground stems [119,120,121,122,123]. The formation of tubers occurs when plants are exposed to the certain conditions, like short days, or when they reach a certain age, and they start to enlarge dramatically upon their initiation [119,123,124,125]. The initiation of potato tuberisation has been well characterised and has been reviewed [119]. Here, we mainly focus on the bulking stage of storage organs and putative approaches to enhance the secondary growth via modulation of the underlying signalling pathways. 

The transverse structure of tubers varies but as a common feature they possess a high number of parenchyma cells for storage [106,126,127,128,129,130,131] (Figure 5B,C). To generate such structure during organogenesis, high cambial activity is needed to increment the number of cells and inhibit the differentiation of the parenchyma cells to the xylem fibres at the same time. This suggests that engineering tubers to reinforce cambial activity and to sustain the cells as parenchyma cells could increase the capacity and/or size of the storage organs. For this, knowledge about xylem differentiation in *Arabidopsis* secondary growth is of great value. One approach to improve cambial activity in the storage organs could be to engineer cytokinin biogenesis or signalling as cytokinins are crucial for cambial activity. Similarly, the CLE41/44/TDIF-PXY/TDR module and WOX4/14 transcription factors play a crucial part in the cambial activity and could be manipulated to enhance cell proliferation. GA is one of the key factors inducing xylem fibre differentiation. Inhibiting GA signalling or the downstream transcription factors, such as NST1 and NST3, could prevent xylem fibre differentiation of parenchyma cells and contribute to increasing storage capacity. Indeed, there are a few studies showing that storage organs development involves molecular components similar to modulators of *Arabidopsis* secondary development. Jang and co-workers showed that in a radish inbred line development of a larger storage organ correlates with stronger cambial activities and higher cytokinin responses in the cambium [106]. They demonstrated that exogenous cytokinin treatment can result in a substantial increase in cell proliferation in the cambium zone and overall secondary growth in a dose-dependent manner, suggesting that cytokinin signalling and responses are crucial for the secondary thickening of radish [106]. In addition, cytokinin signalling seems to be important for the initiation of tuberisation as the overexpression a cytokinin biosynthesis gene in tomato or exogenous cytokinin application together with sucrose of potato lead to the storage organ formation from their axillary buds [119,132]. Gancheva and co-workers showed that the transcripts of *RsCLE41*, the *AtCLE41* homologue, is primarily expressed in the cambium and the phloem of the radish. Interestingly, its expression in *Raphanus sativus* is much higher than in the presumably ancestral *Raphanus raphanistrum*, that does not produce the enlarged taproot tubers [133]. Moreover, exogenous treatment or overexpression of RsCLE41 increases the number of meristematic foci in the centre of the secondary xylem and facilitates cell division in the regular cambium and the meristematic foci. This suggests that the RsCLE41-mediated signalling is involved in the secondary growth of radish as well as in *Arabidopsis* [133]. GA induces xylem fibre differentiation in *Arabidopsis*, so exogenous GA treatment might reduce the tuber productivity, whereas treatment with the GA biosynthesis inhibitor paclobutrazol (PBZ) might elevate it. Several studies examined the effect of exogenous GA and PBZ treatment on storage organ development and showed that GA-treated carrot and radish are inhibited in storage organ secondary thickening whereas PBZ-treated carrot, radish and potato exhibited enhanced thickening [131,134,135,136]. It was shown that the exogenous GA facilitates the xylem differentiation and increases the lignin content in the carrot [131]. In radish, it was shown that PBZ treatment increases the number of cells in the xylem area and the size of xylem vessels [135], suggesting that the suppression GA signalling can be used to increase storage organ productivity. In addition to applying knowledge gained from unravelling the networks regulating secondary growth in *Arabidopsis*, there have been approaches that characterize genome-wide transcriptomic changes during the tuberisation or comparisons between tuberous and non-tuberous roots to understand the bulking processes in the radish, cassava, and sweet potato [120,125,128,137]. Altogether, the application of those advances can contribute to progress in engineering or breeding to enhance tuber productivity.

## 4. Future Perspectives

Our current understanding of secondary growth provides fundamental knowledge to improve wood formation. On the basis of research on *Arabidopsis* secondary growth, engineering of wood formation in tree species has made great progress in the last decade (e.g., [88,93,102]). It is not yet known exactly how storage organs develop a substantial number of xylem parenchyma cells with high sugar or starch content. The *Arabidopsis* hypocotyl and its underlying regulatory network can be very informative for the examination of storage organ regulation and its engineering for higher productivity. A major question is how the switch between differentiation of fibres versus parenchyma cells is regulated in the hypocotyl of *Arabidopsis*, as well as in its Brassiceae relatives with storage root capacity. Insights into secondary growth regulation, storage root development in crops and in potentially new model species, the identification of potential targets for engineering in those, and the development of adjusted methods are particularly relevant, as crop species exhibiting storage roots are currently not compatible with intensive molecular genetics, thus hampering their genetic analysis and efficient bioengineering.
